# (−)-Epigallocatechin-3-Gallate Inhibits Colorectal Cancer Stem Cells by Suppressing Wnt/β-Catenin Pathway

**DOI:** 10.3390/nu9060572

**Published:** 2017-06-03

**Authors:** Yue Chen, Xiao-Qian Wang, Qi Zhang, Jian-Yun Zhu, Yuan Li, Chun-Feng Xie, Xiao-Ting Li, Jie-Shu Wu, Shan-Shan Geng, Cai-Yun Zhong, Hong-Yu Han

**Affiliations:** 1 Department of Nutrition and Food Safety, School of Public Health, Nanjing Medical University, Nanjing 211166, China; chenyue1005@njmu.edu.cn (Y.C.); wangxiaoqian@njmu.edu.cn (X.-Q.W.); qizhang3036@njmu.edu.cn (Q.Z.); njmuzhujy@njmu.edu.cn (J.-Y.Z.); yuanli0321@njmu.edu.cn (Y.L.); xiechunfeng932@njmu.edu.cn (C.-F.X.); xiaotingli@njmu.edu.cn (X.-T.L.); jwu@njmu.edu.cn (J.-S.W.); gss9814@njmu.edu.cn (S.-S.G.); 2 The Key Laboratory of Modern Toxicology, Ministry of Education, School of Public Health, Nanjing Medical University, Nanjing 211166, China; 3 Department of Clinical Nutrition, Sun Yat-sen University Cancer Center, State Key Laboratory of Oncology in South China, Guangzhou 510060, China

**Keywords:** EGCG, colorectal cancer stem cells, Wnt/β-catenin pathway

## Abstract

The beneficial effects of tea consumption on cancer prevention have been generally reported, while (−)-Epigallocatechin-3-gallate (EGCG) is the major active component from green tea. Cancer stem cells (CSCs) play a crucial role in the process of cancer development. Targeting CSCs may be an effective way for cancer intervention. However, the effects of EGCG on colorectal CSCs and the underlying mechanisms remain unclear. Spheroid formation assay was used to enrich colorectal CSCs from colorectal cancer cell lines. Immunoblotting analysis and quantitative real-time polymerase chain reaction were used to measure the alterations of critical molecules expression. Immunofluorescence staining analysis was also used to determine the expression of CD133. We revealed that EGCG inhibited the spheroid formation capability of colorectal cancer cells as well as the expression of colorectal CSC markers, along with suppression of cell proliferation and induction of apoptosis. Moreover, we illustrated that EGCG downregulated the activation of Wnt/β-catenin pathway, while upregulation of Wnt/β-catenin diminished the inhibitory effects of EGCG on colorectal CSCs. Taken together, this study suggested that EGCG could be an effective natural compound targeting colorectal CSCs through suppression of Wnt/β-catenin pathway, and thus may be a promising agent for colorectal cancer intervention.

## 1. Introduction

Colorectal cancer is the third most common cancer and the most common gastrointestinal cancer in the world. As predicted, more than 1 million people will develop colorectal cancer every year [1]. Despite the advancement of major therapeutic strategies including surgery and chemotherapy, the main causes of death are metastasis and recurrence [2]. Accumulating evidence has demonstrated the existence of cancer stem cells (CSCs) in various solid cancer including colorectal cancer [3]. CSCs are a rare subpopulation of cancer cells that exhibit the abilities of self-renewal and multipotent differentiation. CSCs play a key role in tumor initiation and development. CSCs are also crucial for metastasis, drug resistance, as well as recurrence of malignancies [3,4,5,6]. Thus, targeting colorectal CSCs may be an effective approach to preventing the metastasis and recurrence of colorectal cancer.

**Figure 1:**
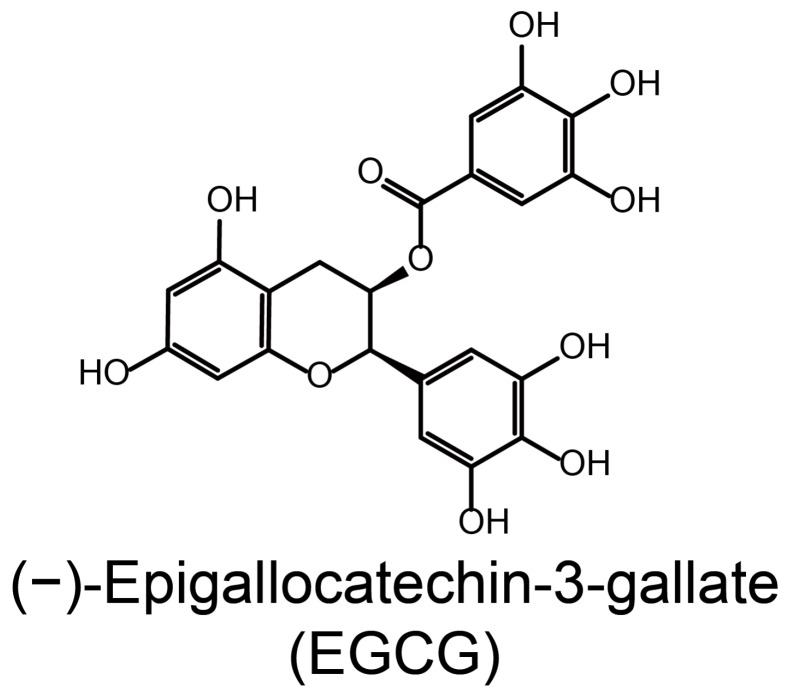
The structure of EGCG.

Wnt/β-catenin pathway is considered one of the most important pathways in the maintenance of CSCs properties [7]. In the absence of Wnt signal, β-catenin is phosphorylated by APC/Axin/GSK3β complex and degraded by proteasome to maintain a low level in cytoplasm. When membrane receptors are activated by Wnt signal, GSK3β is phosphorylated and degraded, resulting in a high level of β-catenin. Accumulated β-catenin then translocates to the nucleus and leads to the activation of target genes such as CD133, CD44, ALDH, c-Myc, and Cyclin D1 [7,8]. As reported, Wnt/β-catenin pathway plays a key role in colorectal CSCs [5]. Mir et al. found that, through reprograming the expression of tumor-associated genes, Wnt/β-catenin pathway promoted colorectal cancer tumorigenesis and progression [9]. 

(−)-Epigallocatechin-3-gallate (EGCG) is the most abundant polyphenol from green tea [10], a widely consumed beverages (Figure 1). Previous studies have shown that the anti-cancer activity of EGCG involves inhibition of proliferation and induction of apoptosis [11]. Furthermore, the inhibition of autophagy and oxidation also participates in the anti-cancer activity of EGCG [12]. Nevertheless, it has been appreciated that EGCG also displays an intriguing effect in adjuvant therapy and prevention of recurrence. As reported, EGCG reduced the recurrence by 51.6% in patients with colorectal adenoma after polypectomy [13]. Combining EGCG with cisplatin or oxaliplatin enhanced the therapeutic effect in human colorectal cancer cells [14]. EGCG exhibited an inhibitory effect on lung CSCs [15]. Considering the tissue-specificity and sensitivity, the inhibitory effects of EGCG on various CSCs may be different. 

Therefore, the present study aimed to investigate the influence of EGCG on colorectal CSCs as well as the underlying mechanisms. Here, we documented that EGCG inhibited colorectal CSCs properties through suppression of Wnt/β-catenin pathway. 

## 2. Materials and Methods

### 2.1. Cell Culture and Reagents 

Human colon cancer cell lines DLD-1 and SW480 were obtained from American Type Culture Collection and maintained at 37 °C in incubator containing 5% CO_2_. Both cell lines were grown in RPMI 1640 medium (Gibco, Carlsbad, CA, USA) supplemented with 10% fetal bovine serum (Gibco, Carlsbad, CA, USA), 100 U/mL penicillin, and 100 mg/mL streptomycin (Gibco, Carlsbad, CA, USA). EGCG and dimethyl sulfoxide were purchased from Sigma (St. Louis, MO, USA). LiCl was acquired from Biosharp (Hefei, China).

### 2.2. Spheroid Formation Assay

DLD-1 and SW480 cells were washed and transferred to DMEM-F12 medium (Gibco, Carlsbad, CA, USA) in non-adherent dishes (Costar). This medium contained no serum but 20 ng/mL EGF (Rocky Hill, NJ, USA), 5 μg/mL insulin (Rocky Hill, NJ, USA), 10 ng/mL bFGF (Rocky Hill, NJ, USA), 0.4% BSA (St. Louis, MO, USA), 2% B27 (Gibco, Carlsbad, CA, USA). The spheroids were grown for six days and fed every 48 h. Spheroids were counted under a microscope (Nikon, Tokyo, Japan) if the diameter was greater than 50 μm. 

### 2.3. Immunoblotting Analysis

Cells were harvested and washed with PBS and lysed with lysis buffer. Concentrations of the protein were determined by the bicinchoninic acid protein assay (Pierce, Rockforsd, WI, USA). Forty micrograms of proteins were subject to 7.5–10% SDS-PAGE and transferred to NC membrane (Millipore, Billerica, MA, USA). After being blocked with 5% non-fat milk for 1 h, the membranes were incubated with relevant primary antibody overnight at 4 °C. Then, the membranes were washed and probed with horseradish peroxidase-conjugated secondary antibody. GAPDH served as the loading control. The antibodies used in the present study were purchased from Proteintech (Rocky Hill, NJ, USA).

### 2.4. Quantitative Real-Time Polymerase Chain Reaction (qRT-PCR)

Total RNA was isolated with Trizol reagent (Invitrogen, Carlsbad, CA, USA). Total RNA (1 μg) was transcribed into cDNA with the 5× All-In-One RT MasterMix (Applied Biosystems, Foster City, CA, USA). The qRT-PCR was performed using an EvaGreen 2× qPCR MasterMix (Applied Biosystems, Foster City, CA, USA) and ABI 7300 real-time PCR detection system (Applied Biosystems, USA). The levels of mRNA expression for each gene were normalized by its respective GAPDH. Fold changes in gene expression were calculated by a comparative threshold cycle (Ct) method using the formula 2^−(ΔΔCt)^. The PCR primers were synthesized by Beijing Genomics Institute (Beijing, China), and the primers used were as follows:

CD133-F, 5′-TACAACGCCAAACCACGACTGT-3′;
CD133-R, 5′-TCTGAACCAATGGAATTCAAGACCCTTT-3′;
CD44-F, 5′-GACACATATTGTTTCAATGCTTCAGC-3′;
CD44-R, 5′-GATGCCAAGATGATCAGCCATTCTGGAAT-3′;
ALDHA1-F, 5′-GCACGCCAGACTTACCTGTC-3′;
ALDHA1-R, 5′-CCTCCTCAGTTGCAGGATTAAAG-3′;
Oct4-F, 5′-TGGGATATACACAGGCCGATG-3′;
Oct4-R, 5′-TCCTCCACCCACTTCTGAG-3′;
Nanog-F, 5′-TTTGTGGGCCTGAAGAAAACT-3′;
Nanog-R, 5′-AGGGCTGTCCTGAATAAGCAG-3′;
β-catenin-F, 5′-AAGACATCACTGAGCCTCCAT-3′;
β-catenin-R, 5′-CGATTTGCGGGACAAAGGGCAA-3′;
PCNA-F, 5′-CTGAAGCCGAAACAGCTAGACT-3′;
PCNA-R, 5′-TCGTTGATGAGGTCTTGAGTGC-3′;
Cyclin D1-F, 5′-AGGCCCTGGCTGCTACAAG-3′;
Cyclin D1-R, 5′-ACATCTGAGTGGGTCTGGAG-3′;
GAPDH-F, 5′-CAAGGTCACCATGACAACTTTG-3′;
GAPDH-R, 5′-GTCCACCACCCTGTTGCTGTAG-3′.

### 2.5. Immunofluorescence Staining

After treatment for six days, the cell spheroids were washed and fixed with paraformaldehyde. The cell spheroids were then blocked with 5% BSA and stained with rabbit CD133 antibody from Proteintech (Rocky Hill, NJ, USA) at 4 °C overnight. After being washed with PBS, the cell spheroids were stained with FITC-conjugated goat-anti-rabbit antibody (Rocky Hill, NJ, USA) for 1 h. The cell spheroids were then counterstained with 4,6′-diamidino-2-phenylindole (DAPI) for 10 min. The fluorescent images were obtained using a fluorescence microscope (Olympus, Tokyo, Japan).

### 2.6. Statistical Analysis

Data are expressed as means ± SD. Student’s *t*-test and one-way analysis of variance (ANOVA) were performed to compare the difference between two or multiple groups. Significant difference was taken as * *p* < 0.05 or ** *p* < 0.01. All analyses were performed with SPSS version 11.0 software.

## 3. Results

### 3.1. Enrichment of Colorectal CSCs by Serum-Free-Medium Culture

**Figure 2:**
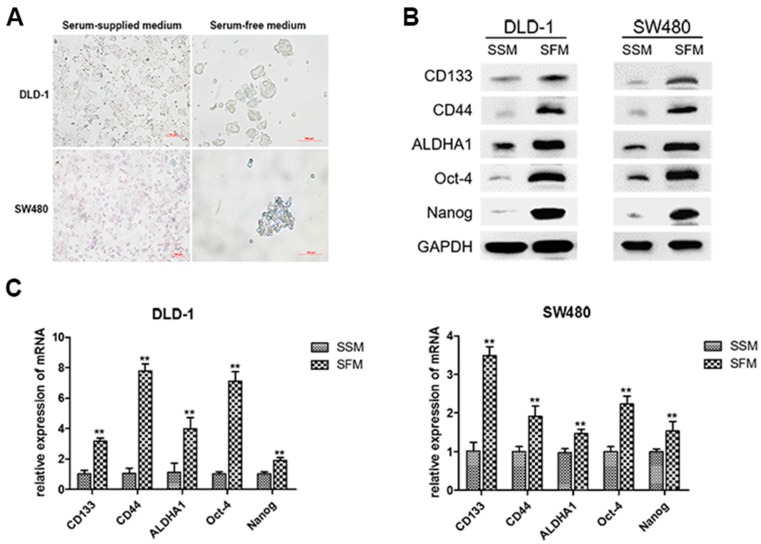
Enrichment of colorectal CSCs by serum-free-medium culture. DLD-1 and SW480 cells were cultured in serum-supplied medium (SSM) and serum-free medium (SFM) for six days. (**A**) Cell morphology was imaged under a light microscope. (**B**) The protein levels of CSC markers, including CD133, CD44, ALDHA1, Nanog, and Oct4, were measured by Western blotting analysis. (**C**) The mRNA levels of corresponding CSC markers were examined by Quantitative real-time PCR. Data are expressed as mean ± SD of three independent experiments. ** *p* < 0.01 compared with control group.

Accumulating evidence has revealed that spheroids formed in serum-free medium (SFM) exhibited more stem cell properties [16]. CD133, CD44, ALDHA1, Oct-4, and Nanog were reported to be the key CSC markers in colorectal cancer [17]. As shown in Figure 2A, SFM culture led to spheroid formation of DLD-1 and SW480 colorectal cancer cells. After cultured in SFM for six days, the protein levels of colorectal CSC markers were dramatically increased (Figure 2B). Similarly, qRT-PCR analysis showed that the mRNA levels of the pivotal CSC markers were also upregulated in both cell lines (Figure 2C). These results suggested the characteristics of colorectal CSCs in DLD-1 and SW480 sphere-forming cells cultured in SFM.

### 3.2. EGCG Inhibited the CSCs Properties and Wnt/β-Catenin Pathway in Colorectal CSCs

**Figure 3:**
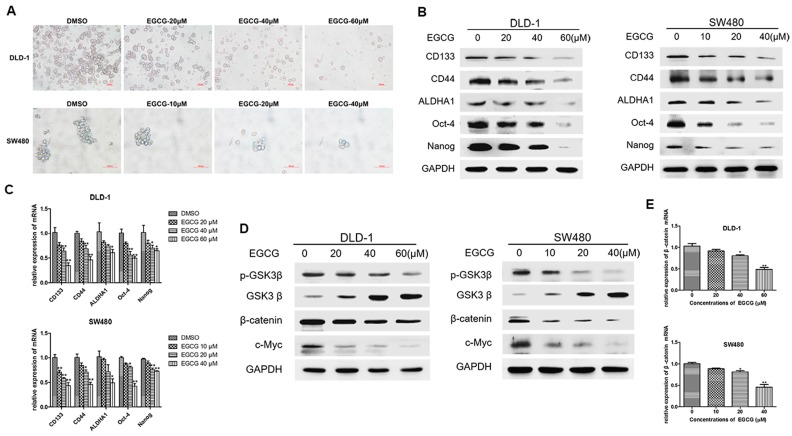
EGCG inhibited the CSCs properties and Wnt/β-catenin pathway in colorectal CSCs. DLD-1 and SW480 cells were treated with different concentrations of EGCG for six days. (**A**) Images of DLD-1 and SW480 spheroids; (**B**) The protein levels of colorectal CSC markers, including CD133, CD44, ALDHA1, Nanog, and Oct4, were measured by Western blotting analysis; (**C**) The corresponding mRNA levels were measured by Quantitative real-time PCR; (**D**) The protein levels of Wnt/β-catenin pathway (p-GSK3β, GSK3β, β-catenin, and c-Myc) were determined by Western blot analysis; (**E**) Quantitative real-time PCR analysis for the mRNA levels of β-catenin. Data are expressed as mean ± SD of three independent experiments. * *p* < 0.05, ** *p* < 0.01 compared with control group.

We next examined the inhibitory effects of EGCG on colorectal cancer spheroids. DLD-1 and SW480 cell spheroids were treated with different concentrations of EGCG for six days. Spheroid formation assay showed that EGCG treatment led to a significant decrease in the number and size of DLD-1 and SW480 cell spheroids in a dose dependent manner (Figure 3A). Western blot analysis showed that the protein levels of colorectal CSC markers were significantly reduced in the spheroids of both cell lines (Figure 3B). Meanwhile, the mRNA levels of CSC markers showed similar changes with the proteins (Figure 3C). Considering the critical role of Wnt/β-catenin pathway in CSCs, we next investigated whether EGCG could modulate Wnt/β-catenin pathway in colorectal CSCs. We revealed that EGCG downregulated the expression of p-GSK3β (Ser-9), upregulated the expression of GSK3β (the key negative regulator of Wnt/β-catenin pathway), and decreased the level of β-catenin as well as its target gene c-Myc (Figure 3D). As shown in Figure 3E, the mRNA expression of β-catenin was decreased in DLD-1 and SW480 cell spheroids. These results illustrated that EGCG inhibited colorectal CSC properties as well as Wnt/β-catenin pathway in DLD-1 and SW480 cell spheroids.

### 3.3. EGCG Reduced Cell Proliferation and Induced Apoptosis of Colorectal CSCs

**Figure 4:**
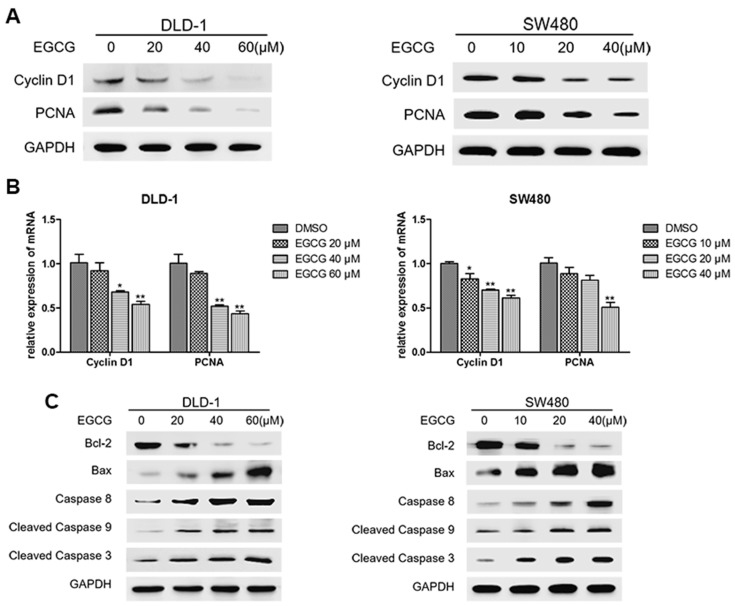
EGCG reduced cell proliferation and induced apoptosis of colorectal CSCs. DLD-1 and SW480 spheroids were treated with different concentrations of EGCG for six days. (**A**) Protein expression levels of PCNA and Cyclin D1 were measured by Western blot analysis; (**B**) Quantitative real-time PCR analysis for the detection of mRNA levels of PCNA and Cyclin D1; (**C**) Expression levels of apoptosis related proteins, including Bcl-2, Bax, Caspase 8, Cleaved Caspase 9, and Cleaved Caspase 3, were measured by Western blot analysis. Data are expressed as mean ± SD of three independent experiments. * *p* < 0.05, ** *p* < 0.01 compared with control group.

To further investigate the effect of EGCG on spheroid growth in vitro, we next evaluated the alteration in cell proliferation as well as apoptosis after EGCG treatment. As shown in Figure 4A, EGCG treatment resulted in significant decrease in the protein levels of Cyclin D1 and PCNA. In addition, qRT-PCR analysis showed that the mRNA levels of Cyclin D1 and PCNA were also decreased (Figure 4B). Meanwhile, EGCG treatment also downregulated the expression of Bcl-2 and upregulated the levels of Bax, Caspase 8, Caspase 9, and Caspase 3 (Figure 4C). Together, these results suggested that EGCG reduced cell proliferation and induced apoptosis of colorectal CSCs.

### 3.4. EGCG Diminished Colorectal CSCs Properties through Suppression of Wnt/β-Catenin Pathway

**Figure 5:**
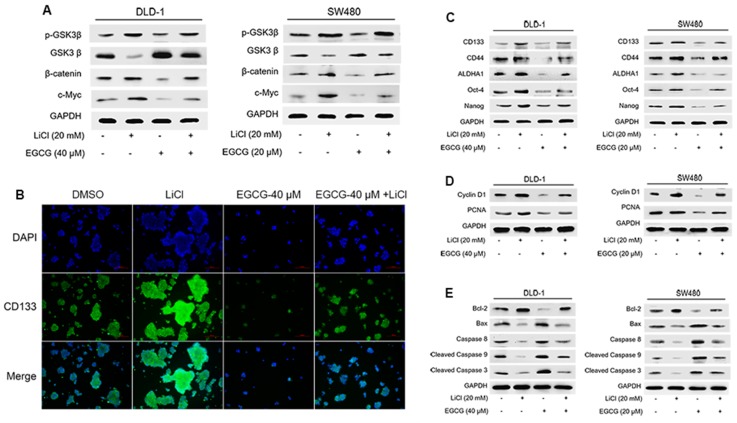
EGCG diminished colorectal CSCs through suppression of Wnt/β-catenin pathway. DLD-1 and SW480 spheroids were treated with different concentrations of EGCG and LiCl for six days. (**A**) Western blot analysis was applied to measure the protein levels of Wnt/β-catenin pathway; (**B**) Immunofluorescence staining images of spheroids were obtained to determine the expression of CD133. (**C**–**E**) Western blot analysis was used to measure the protein levels of CSC markers (**C**), proliferation-related proteins (**D**), and apoptosis-related proteins (**E**).

To determine the role of Wnt/β-catenin pathway in the inhibitory effects of EGCG on colorectal CSCs, LiCl was applied to activate Wnt/β-catenin pathway. As predicted, Figure 5A shows that EGCG treatment again downregulated Wnt/β-catenin pathway, whereas LiCl activated it. Notably, co-treatment of LiCl and EGCG reversed the inhibitory effect of EGCG on Wnt/β-catenin pathway. Immunofluorescence staining analysis also indicated that LiCl diminished the inhibitory effects of EGCG on spheroid formation and CD133 expression (Figure 5B). These results were consistent with Western blot analysis, which showed that the effects of EGCG on colorectal CSC markers expression (Figure 5C), cell proliferation (Figure 5D), and apoptosis-related proteins (Figure 5E) were abrogated by LiCl-triggered Wnt/β-catenin activation. Together, these data revealed that EGCG inhibited colorectal CSC properties through suppression of Wnt/β-catenin pathway.

## 4. Discussion

With the acknowledgement of cancer stem cell theory, the critical role of CSCs in cancer development, progression, and drug resistance has been demonstrated. Wnt/β-catenin pathway is considered as the vital pathway in maintaining the stemness of CSCs. EGCG, a natural compound from green tea, is identified as a chemopreventive and chemotherapeutic agent for cancer. Herein, we revealed that EGCG inhibited colorectal CSCs through targeting Wnt/β-catenin pathway. These results may provide new insights into the interventional strategies against colorectal CSCs. 

In the present study, we enriched colorectal CSCs from adherent colorectal cancer cell lines DLD-1 and SW480 using a serum-free-medium stem cell culture system, one of the commonly used methods for the isolation and enrichment of CSCs which is based on CSCs capability to form three-dimensional tumorspheres in vitro. We showed the tumorsphere formation capability of both DLD-1 and SW480 cells cultured in SFM. Meanwhile, the enrichment effect was verified by the changes of the protein and mRNA expression levels of colorectal CSC markers, including CD133, CD44, ALDHA1, Oct-4, and Nanog. CD133, CD44, and ALDHA1 were considered as the CSC markers in colorectal cancer [5,6]. In addition, Oct-4 and Nanog were recognized as ‘stem cell genes’ since the induction of stem cells in mice was demonstrated by the milestone research of Professor Shinya Yamanaka. Our results showed markedly elevated levels of these distinct colorectal CSC markers in the sphere-forming cells. These data depicted the CSC characteristics of these cells cultured in stem cell-conditional medium in vitro. 

Since CSCs are considered the driving force of cancer initiation, development, and drug resistance, great efforts have been made to develop chemical approaches targeting CSCs [18,19]. Strikingly, many natural products have been reported as active against CSCs [20]. For example, sulforaphane was reported to inhibit the expression of pluripotency maintaining transcription factors and self-renewal of pancreatic CSCs [21]. Curcumin was shown to be able to regulate self-renewal pathways and target CSCs in breast and lung cancer [22,23]. Resveratrol effectively killed ovarian CSCs independent of reactive oxygen species [24]. Quercetin suppressed the expression of CSC markers in pancreatic cancer stem-like cells [25]. Retinoic acid was demonstrated to target glioblastoma stem cells through Notch pathway [26]. Investigation of more natural products against CSCs and the underlying mechanisms will provide novel information regarding the potential preventive and therapeutic application of these compounds against CSCs [27]. A number of epidemiologic studies have illustrated the cancer-preventive effects of green tea [28,29], among which EGCG is the most abundant polyphenol. Accumulating evidence has shown that EGCG exhibits anti-cancer properties in various types of cancer—including bladder cancer [30], melanoma [31] and lung cancer [32]—and its anti-cancer mechanisms involve inhibition of proliferation [33] and induction of apoptosis [34]. Recent evidence suggested that EGCG hindered human colon cancer sphere formation [35]. Toden et al. found that EGCG targeted cancer stem-like cells and enhanced 5-fluorouracil chemosensitivity in colorectal cancer, and this effect was accompanied by inhibition of Notch pathway; however, the role of Notch pathway in EGCG suppression of colorectal CSCs was not demonstrated yet [36]. Therefore, the underlying mechanisms of EGCG suppression of colorectal CSCs remain to be elucidated. Here, we demonstrated that suppression of Wnt/β-catenin pathway, another key CSCs regulatory signaling, mediated the inhibitory effects of EGCG on colorectal CSCS. We showed in the present study that EGCG treatment led to suppression of Wnt/β-catenin pathway in colorectal CSCs, while LiCl-triggered activation of Wnt/β-catenin pathway abrogated the inhibitory effects of EGCG. Thus, our study illustrated that EGCG inhibited colorectal CSCs through targeting Wnt/β-catenin pathway-a finding that has not been previously reported in colorectal CSCs. These results provided further information in understanding the inhibitory effects of EGCG on colorectal CSCs.

Wnt/β-catenin pathway is a highly conserved signaling, participating in embryonic development, homeostasis maintenance, and cancer development as well. It has been shown that in colorectal cancer, about 80% of tumors have nuclear accumulation of β-catenin and 90% are endowed with an activated level of Wnt/β-catenin pathway [37]. Wnt/β-catenin pathway is also pivotal for the properties of CSCs. Increased nuclear β-catenin level was observed in imatinib-resistant patients with chronic myelogenous leukemia [38]. Wnt/β-catenin activity regulated the self-renewal property and proliferation of stem-like prostate cancer cells in an androgen-independent manner [39]. It has been noted that EGCG regulates Wnt/β-catenin pathway. The anti-adipogenic effects of EGCG were dependent on Wnt/β-catenin pathway [40]. EGCG exerted its anticancer activity by promoting the phosphorylation and proteasomal degradation of β-catenin through a mechanism independent of the GSK-3β and PP2A [41]. We showed in our study that EGCG treatment led to a downregulation of Wnt/β-catenin pathway, while LiCl-triggered activation of Wnt/β-catenin pathway abrogated the inhibitory effects of EGCG on spheroid formation, CSC markers expression, cell proliferation, and apoptosis of colorectal CSCs. Taken together, these data illustrated that EGCG inhibited colorectal CSCs through the suppression of Wnt/β-catenin pathway.

## 5. Conclusions

In conclusion, our study suggested that EGCG inhibited colorectal CSCs through targeting Wnt/β-catenin pathway. These results may provide a new vision on exploring natural compound as well as new strategies for cancer intervention. It should be noted that the inhibitory effects of EGCG on various CSCs may be different, and hence more studies are warranted to evaluate its efficacy in different types of cancers.
